# Unveiling the therapeutic potential: KBU2046 halts triple-negative breast cancer cell migration by constricting TGF-β1 activation *in vitro*

**DOI:** 10.32604/or.2024.049348

**Published:** 2024-10-16

**Authors:** JINXIA CHEN, SULI DAI, GENG ZHANG, SISI WEI, XUETAO ZHAO, YANG ZHENG, YAOJIE WANG, XIAOHAN WANG, YUNJIANG LIU, LIANMEI ZHAO

**Affiliations:** 1 Research Center, The Fourth Hospital of Hebei Medical University, Shijiazhuang, 050000, China; 2 Key Laboratory of Tumor Gene Diagnosis, Prevention and Therapy, Clinical Oncology Research Center, Shijiazhuang, 050000, China; 3 Department of Blood Transfusion, The Fourth Hospital of Hebei Medical University, Shijiazhuang, 050000, China; 4 Breast Center, Fourth Hospital of Hebei Medical University, Shijiazhuang, 050000, China; 5 Hebei Provincial Key Laboratory of Tumor Microenvironment and Drug Resistance, Hebei Medical University, Shijiazhuang, 050000, China

**Keywords:** KBU2046, TGF-β1 (transforming growth factor-β1), LRRC (leucine-rich repeat-containing), LTBP (leucine-rich repeat-containing), Breast cancer (BC), Integrin αv, Integrin α6

## Abstract

**Background:**

Triple-negative breast cancer (TNBC) is a heterogeneous, recurring cancer characterized by a high rate of metastasis, poor prognosis, and lack of efficient therapies. KBU2046, a small molecule inhibitor, can inhibit cell motility in malignant tumors, including breast cancer. However, the specific targets and the corresponding mechanism of its function remain unclear.

**Methods:**

In this study, we employed (3-(4,5-dimethylthiazol-2-yl)-5-(3-carboxymethoxyphenyl)-2-(4-sulfophenyl)-2H tetrazolium) (MTS) assay and transwell assay to investigate the impact of KBU2046 on the proliferation and migration of TNBC cells *in vitro*. RNA-Seq was used to explore the targets of KBU2046 that inhibit the motility of TNBC. Finally, confirmed the predicted important signaling pathways through RT-qPCR and western blotting.

**Results:**

In this study, we found that KBU2046 functioned as a novel transforming growth factor-β (TGF-β1) inhibitor, effectively suppressing tumor cell motility *in vitro*. Mechanistically, it directly down-regulated leucine-rich repeat-containing 8 family, member E (LRRC8E), latent TGFβ-binding protein 3 (LTBP3), dynein light chain 1 (DNAL1), and MAF family of bZIP transcription factors (MAFF) genes, along with reduced protein expression of the integrin family. Additionally, KBU2046 decreased phosphorylation levels of Raf and ERK. This deactivation of the ERK signaling pathway impeded cancer invasion and metastasis.

**Conclusions:**

In summary, these findings advocate for the utilization of TGF-β1 as a diagnostic and prognostic biomarker and as a therapeutic target in TNBC. Furthermore, our data underscore the potential of KBU2046 as a novel therapeutic strategy for combating cancer metastasis.

## Introduction

Tumor metastasis stands as the primary contributor to treatment failure in cancer patients, constituting approximately 90% of tumor-related mortality [1]. The process of metastasis involves intricate signaling pathways and communication among diverse cell types [2]. Breast cancer, the most prevalent malignancy, stands as the leading cause of cancer-related fatalities in women. Triple-negative breast cancer (TNBC), distinguished by the absence of human epidermal growth factor receptor 2 and hormone receptors, comprises approximately 15%–20% of all breast cancers [3]. TNBC, characterized by a lack of effective treatments, demonstrates the highest mortality rate among breast cancers, primarily attributed to metastasis [4,5]. Consequently, there is an imperative need to uncover the molecular mechanisms governing the tumorigenesis and metastasis of TNBC and subsequently devise effective anti-cancer drugs.

Recent studies have underscored the efficacy of small molecule inhibitors, such as KBU2046 [6], Nivolumab [7], SB-3CT [8], and 264RAD [9], in suppressing tumor metastasis and progression. Notably, KBU2046 demonstrates the ability to inhibit cell movement without inducing cytotoxicity, making it the most promising therapeutic candidate [6]. Derived from 4,5,7-trihydroxy isoflavone (genistein) due to its established anti-motility properties [6], KBU2046 stands out among these inhibitors. However, its detailed mechanism and targets of action remain unclear.

Transforming growth factor β (TGF-β) encompasses a group of cytokines with pleiotropic effects on cell migration, differentiation, immune regulation, and the induction of epithelial-mesenchymal transition [10]. These cytokines play a crucial role in various diseases, including cancer, fibrosis, and neurodegenerative diseases, attributed to aberrations in the TGF-β signaling pathway [11]. Initially produced as an inactive precursor form, known as latent TGF-β (L-TGF-β) [12], TGF-β combines with several anchor proteins during secretion. Two major protein families, latent TGF-β binding proteins (LTBPs) and leucine-rich repeat-containing (LRRC) 32/33 proteins, directly crosslink with the latent form of TGF-β. The crosslinking occurs in a cell type-specific or biological context-dependent manner [13]. Activation of L-TGF-β ensues through various mechanisms, with the most significant being mediated by integrins αvβ6 and αvβ8 [14–16]. Consequently, KBU2046 exerts its anti-tumor migration activity by impeding the maturation of TGF-β1.

In summary, we utilized mRNA-seq analysis and Western blotting to identify novel targets for TNBC and elucidated the mechanism by which KBU2046 inhibits cellular motility. This inhibitory effect was achieved by decreasing the expression of LTBP and LRRC at the mRNA level and reducing the expression of the integrin family at the protein level. These findings underscore TGF-β1 as a promising therapeutic target for TNBC and position KBU2046 as a novel approach in cancer treatment.

## Materials and Methods

### Cell culture

The TNBC cell lines, BT-549 and MDA-MB-231, were procured from the ATCC cell bank. BT-549 cells (ATCC, VA, USA) were maintained in RPMI-1640 medium (C11875500BT, Gibco, CA, USA) supplemented with 10% heat-inactivated fetal bovine serum (FBS) (10091-148, Gibco, CA, USA) and 1% penicillin/streptomycin (P0781, Sigma-Aldrich, MO, USA) at 37°C in a humidified atmosphere containing 5% CO_2_. MDA-MB-231 cells (ATCC, VA, USA) were cultured in DMEM (C11995500BT, Gibco, CA, USA) supplemented with 10% heat-inactivated FBS and 1% penicillin/streptomycin. Throughout the study, all cell lines underwent cytogenetic identification and authentication every 6 months to ensure reliability and consistency.

### Reagents and antibodies

KBU2046 (C_15_H_11_FO_2_, purity ≥96%) was procured from AdipoGen Life Sciences (cat. no. AG-CR1-0159, SD, USA). It was dissolved in dimethyl sulfoxide (D8371, Sigma-Aldrich, MO, USA) at a concentration of 100 mM and stored at −80°C. For experimentation, it was further diluted in RPMI-1640 medium to a final concentration of 1 mM. Recombinant human TGF-β1 was acquired from BioLegend, Inc. (cat. no. #781804, SD, CA). TGF-β1 was dissolved in 1% BSA (V900933, Sigma-Aldrich, MO, USA) at a concentration of 100 µg/mL and stored at −80°C. Before use, it was diluted to a final concentration of 1 µg/mL in RPMI-1640 medium. Cells were incubated in RPMI-1640 or DMEM supplemented with 10% FBS and 1% penicillin/streptomycin until reaching 80% confluence prior to treatment with KBU2046 or mature TGF-β1. Subsequently, cells were exposed to KBU2046 or TGF-β1 for a specified duration in the absence of FBS.

Antibodies used included integrin αv (cat. no. 27096-1-AP, Wuhan, China), integrin α6 (cat. no. 27189-1-AP, Wuhan, China), integrin α3 (cat. no. 21992-1-AP, Wuhan, China), and integrin β8 (cat. no. abs105044, Shanghai, China) obtained from Proteintech Group, Inc. ERK1/2 (cat. no. #4695, SD, USA), p-ERK1/2 (Thr202/Tyr204) (#9101, SD, USA), Raf1 (#9422, SD, USA), and p-Raf1 (Ser338) (#9427, SD, USA) were sourced from Cell Signaling Technology, Inc.

### MTS assay

The cytotoxicity of KBU2046 was assessed using the (3-(4,5-dimethylthiazol-2-yl)-5-(3-carboxymethoxyphenyl)-2-(4-sulfophenyl)-2H tetrazolium) (MTS) assay. A total of 2,000 cells were seeded per well in a 96-well plate (NEST, Wuxi, China) and incubated overnight at 37°C. KBU2046 at concentrations of 1, 5, and 10 μM, diluted in medium, was added to the respective wells, followed by a 48-h incubation at 37°C with 5% CO_2_. For the MTS assay, 20 μL of MTS substrate (G109A, Promega, Wisconsin, USA) was added to each well and incubated for 90 min. Absorbance at a wavelength of 492 nm was measured using a microplate reader (multiskan ascent, Thermo, Shanghai, China). The experiment was conducted in triplicate and repeated three times for robustness.

### Transwell assay

The transwell assay was conducted following established procedures. Briefly, 1 × 10^5^ tumor cells in 200 μL of serum-free RPMI-1640 medium were seeded in the top chamber (Corning Incorporated, NY, USA) of a transwell plate, and 600 μL of RPMI-1640 medium containing 5, 10, and 20 ng/mL TGF-β1 was added to the bottom chamber. Subsequently, 1, 5, and 10 μM of KBU2046 were introduced to the top chamber. After a 24-h incubation, the membranes were fixed with paraformaldehyde and stained with 1% crystal violet (G1062, Solarbio Science & Technology, Beijing, China). Cellular observations were made under a microscope (Ti2-U Nikon eclipse, Tokyo, Japan) and cell counts were conducted in five randomly selected fields.

### RNA extraction and mRNA-seq assay

For the mRNA-seq assay, samples were forwarded to Sinotech Genomics Co., Ltd., located in Shanghai, China, for RNA-seq analysis. Total RNA extraction was performed using the Trizol reagent (15596018, Ambion, Life Technologies, Carlsbad, California, USA). The Qubit^®^3.0 Fluorometer (Life Technologies, USA) and the Nanodrop One spectrophotometer (Thermo Fisher Scientific Inc., USA) were employed to assess the concentration and quality of purified RNA, respectively. The Agilent 2100 Bioanalyzer (Agilent Technologies Inc., USA) was used to evaluate the integrity of total RNA, and only samples with RNA integrity number (RIN) values above 7.0 were considered suitable for sequencing.

Subsequent to RNA extraction, reverse transcription was employed to synthesize cDNA. Clusters were generated by cBot, with the library diluted to 10 pM, and sequencing was performed using the Illumina NovaSeq 6000 (Illumina, USA). Gene Ontology (GO) and Kyoto Encyclopedia of Genes and Genomes (KEGG) analyses were executed using the enrich R package (version 3.4.3). All of the raw data is in the GEO repository as GSE253364.

### Quantitative real-time PCR (qRT-PCR)

RNA was isolated from cells using Trizol reagent, followed by reverse transcription to cDNA with a Reverse Transcription Kit (PRA5001, Promega, USA). Subsequently, the cDNA underwent PCR amplification employing GO Taq qPCR Master Mix (A6002, Promega, Wisconsin, USA). In a 20 μL reaction system, primers with optimal concentrations were added, and PCR detection was carried out using the cDNA obtained from reverse transcription as a template. The expression levels of human LRRC8E, LTBP3, MAFF and DNAL1 were normalized to that of β-actin, and the relative expression of the target genes was calculated using the 2^–ΔΔCT^ method. Table A1 lists the primers used in the qPCR analysis.

### Western blotting analysis

Briefly, 3 × 10^5^ cells were seeded into a 6-well plate (NEST, Wuxi, China) and incubated overnight at 37°C. Subsequently, instead of 1, 5, and 10 μM of KBU2046 were added to the respective wells, and the plates were incubated for 48 h in a 37°C incubator containing 5% CO_2_. Following incubation, cells were harvested by washing twice with pre-cooled PBS. The harvested cells were lysed in RIPA buffer (R0010, Solarbio, Beijing, China) containing protease and phosphorylase inhibitors. Subsequently, equal amounts of total proteins (50 μg) were subjected to sodium dodecyl-sulfate polyacrylamide gel electrophoresis (SDS-PAGE) on a 10% gel and then transferred to a PVDF membrane (Millipore, Massachusetts, USA). The membrane was blocked with skimmed milk at room temperature for 1 h and exposed to primary antibodies against integrin αv, integrin α6, integrin α3, integrin β8, ERK1/2, p-ERK1/2 (Thr202/Tyr204), Raf1, and p-Raf1 (Ser338) (diluted at 1:1,000) at 4°C overnight. Subsequently, the membrane was incubated with the secondary antibody IRDye 800CW Goat anti-Rabbit IgG Secondary Antibody (926-32211, LI-COR, USA) for 1 hour in dark and the antigen-antibody reaction was visualized by detection with an Odyssey assay (Millipore, Billerica, Massachusetts, USA). GAPDH (ab9485, Abcam, Cambridge, UK) was used as the loading control.

### Statistical analysis

GraphPad Prism (GraphPad, version 8.0) and SPSS (IBM, version 22.0) were utilized for all data analyses. Results were presented as mean ± standard deviation (SD). Inter-group comparisons were performed using the student’s *t*-test and Cox Regression. Significance levels were indicated by *p-*values, with *p < 0.05, **p < 0.01 and ***p < 0.001 considered statistically significant. Importantly, all experiments were carried out in triplicate to ensure robustness and reliability.

## Results

### KBU2046 significantly diminishes TNBC cell motility induced by mature TGF-β1, and at specific concentrations, it does not manifest cytotoxic effects

**Figure 1:**
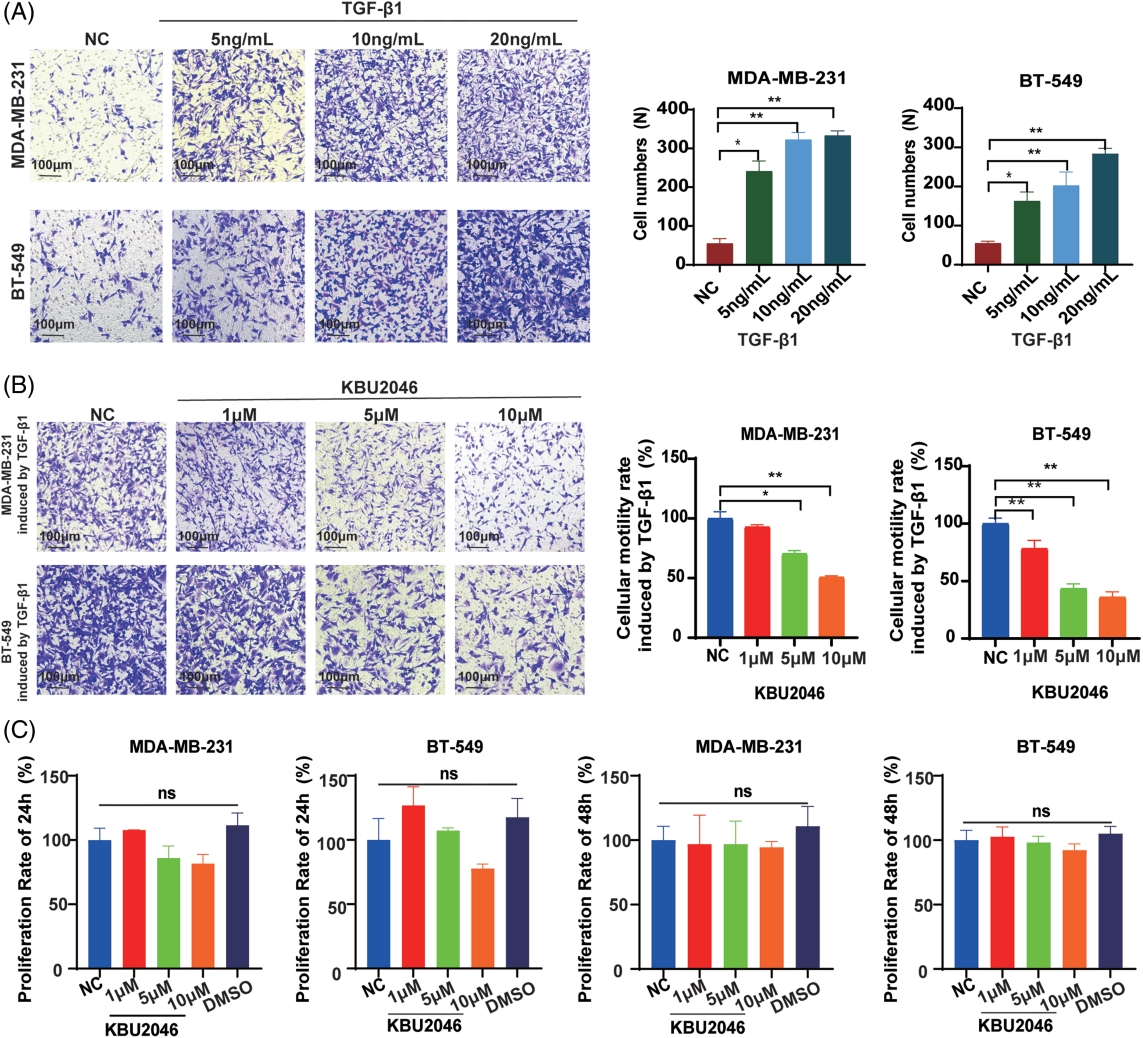
Effects of KBU2046 on breast cancer cells. (A) The effect of different concentrations of mature TGF-β1 on the motility of breast cancer cells evaluated by transwell assay. (B) Inhibitory effect of different concentrations of KBU2046 on motility of breast cancer cells evaluated by transwell assay. (C) The effect of different concentrations of KBU2046 on the proliferation of breast cancer cells detected by MTS assay. DMSO was used as a solvent control. Data are presented as means ± SEM of at least three independent experiments. *p < 0.05; **p < 0.01; ns, no significance.

In our previous studies, KBU2046 has demonstrated effective inhibition of cellular motility in various tumors both *in vivo* and *in vitro* [6]. To validate the impact of mature TGF-β1 on TNBC cell motility, we conducted a transwell assay. As depicted in Fig. 1A, an increase in TGF-β1 concentration corresponded to an elevated number of migrating cells. Our research suggested that TGF-β1 was associated with cellular motility and might serve as a potential target in cancer therapy. Subsequently, we investigated the inhibitory effect of KBU2046 on TGF-β1. As illustrated in Fig. 1B, in MDA-MB-231 cells, at KBU2046 concentrations of 1, 5, and 10 μM the corresponding cellular motility rates were (92.97 ± 1.42)%, (70.59 ± 2.05)%, and (50.77 ± 0.96)%, respectively, while the rate in the reagent control group was (100 ± 4.586)%; in BT-549 cells, the corresponding rates for the same concentrations of KBU2046 were (78.43 ± 5.71)%, (43.55 ± 3.23)%, and (36.09 ± 3.71)% compared to (100 ± 3.91)% in the control group. These results indicated a significant inhibition of TGF-β1-induced cellular motility in TNBC by KBU2046.

To further evaluate the impact of KBU2046 on TNBC cell proliferation, MDA-MB-231 and BT-549 cells were exposed to KBU2046 at concentrations of 1, 5, and 10 μM for 24 and 48 h, followed by MTS assay. No time-or concentration-dependent alterations in cell proliferation were observed (Fig. 1C). Taken together, these findings suggested that KBU2046 robustly suppressed TNBC cellular motility without exerting cytotoxic effects within certain concentrations. The results collectively indicated that KBU2046 held promise as a potential anticancer agent with a favorable safety profile.

### TGFB1 is up-regulated in TNBC and associated with tumor staging and overall survival

**Figure 2:**
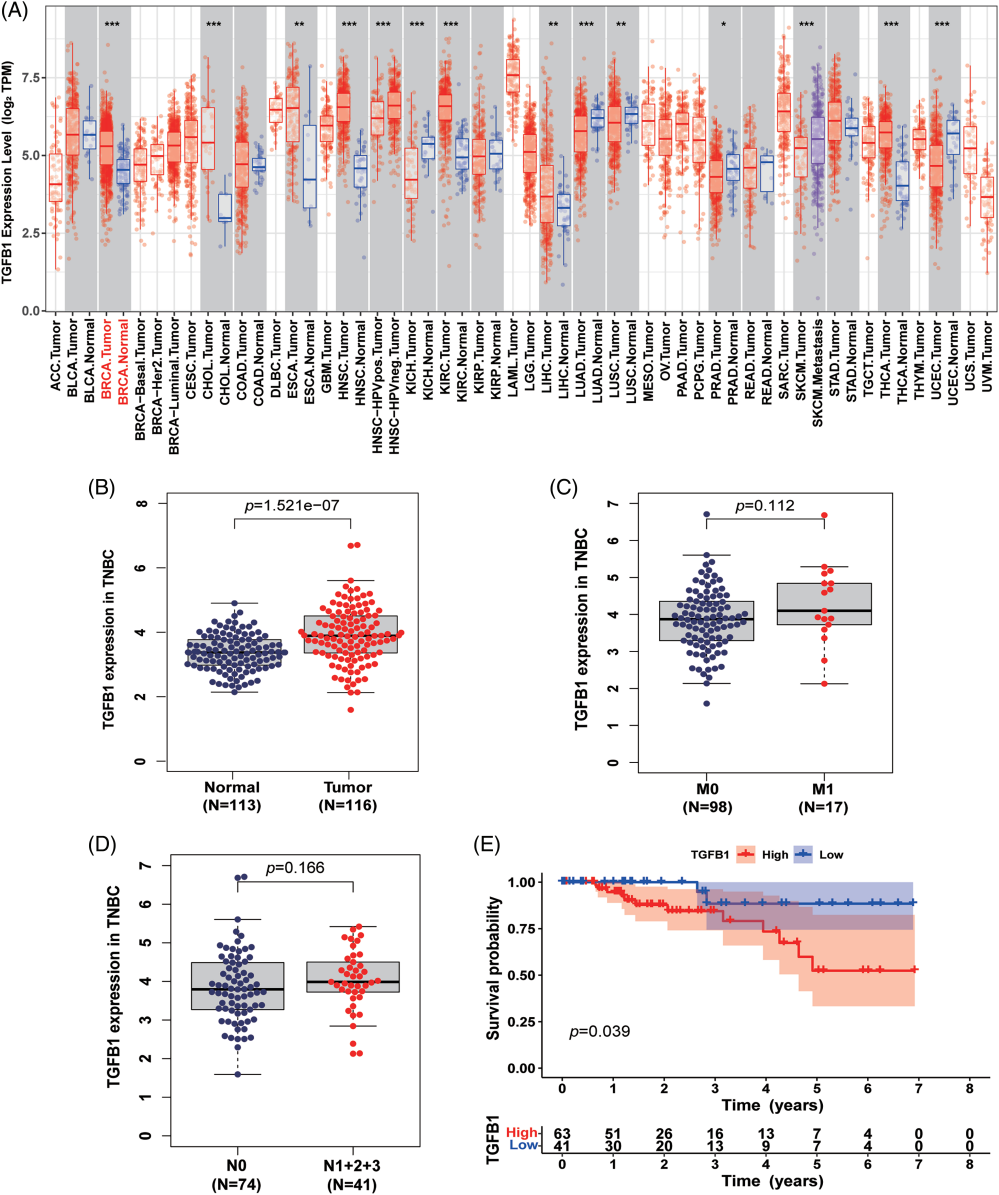
TGF-B1 expression in breast cancer. (A) TGF-β1 was highly up-regulated in breast cancer and associated with cellular motility. Expression levels of TGF-β1 in various cancers from the TCGA database determined by TIMER (*p < 0.05, **p < 0.01, ***p < 0.001). (B) TGF-β1 expression in TNBC from the TCGA database. (C and D) TGF-β1 expression in different M and N stages. (E) The relationship between TGF-β1 expression level and survival.

To assess the differential expression of TGFB1 between primary tumors and matched adjacent nonneoplastic tissues across all TCGA specimens, we utilized the Tumor IMmune Estimation Resource (TIMER2.0) database (https://cistrome.shinyapps.io/timer/). The results revealed an up-regulation of TGFB1 expression in various tumors, including breast cancer, cholangiocarcinoma, and esophageal carcinoma (Fig. 2A). Additionally, we investigated TGFB1 expression in TNBC using the TCGA dataset, observing a notably high expression level (Fig. 2B). Furthermore, the trend of higher TGFB1 expression correlating with lower survival probability and TNM stage in TNBC was consistently observed (Fig. 2C–E). Although statistical significance was not achieved due to the limited number of cases, the trends were evident.

### mRNA-seq analysis is used to explore the inhibitory effect of KBU2046 on cellular motility

**Figure 3:**
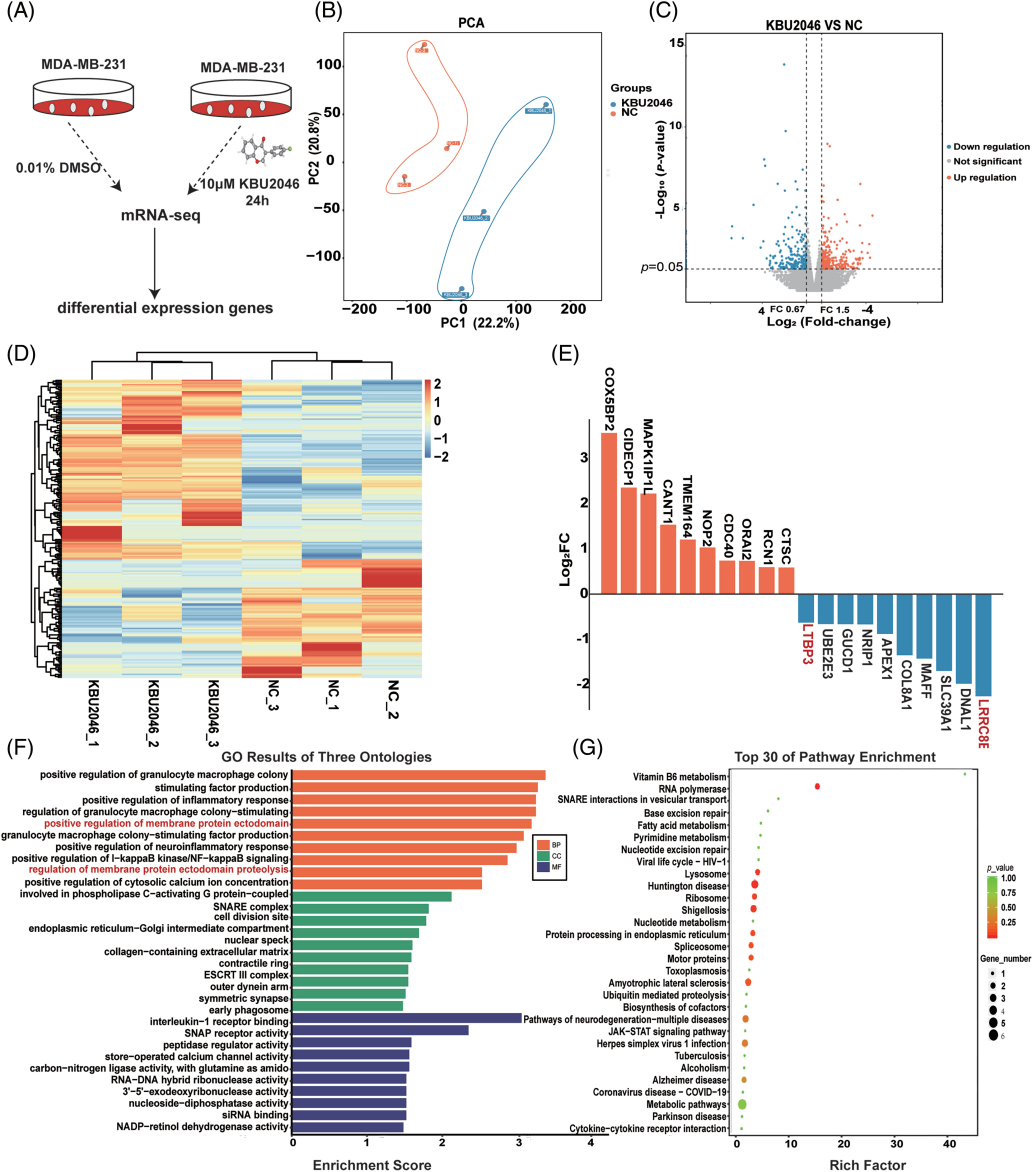
mRNA-seq analysis of KBU2046-treated MDA-MB-231 cells. (A) Workflow illustrating the identification of KBU2046-regulated genes by mRNA-seq analysis. (B) PCA of KBU2046-treated and untreated samples of MDA-MB-231 cells. (C) Volcano diagram for differentially expressed genes after KBU2046 treatment in MDA-MB-231 cells. (D) Cluster analysis of differentially expressed genes after KBU2046 treatment in MDA-MB-231 cells. (E) Top 10 significantly up-regulated and down-regulated genes in the KBU2046-treated group. (F) GO enrichment analysis of differentially expressed genes in the KBU2046-treated group of MDA-MB-231 cells. (G) KEGG pathway analysis of differentially expressed genes in the KBU2046-treated group of MDA-MB-231 cells.

To delve deeper into the mechanism underlying KBU2046’s inhibition of cellular motility, we conducted mRNA-seq analysis to compare changes in the mRNA profiles of KBU2046-treated MDA-MB-231 cells and untreated controls after 24 h of treatment as shown in Fig. 3A. Principal component analysis (PCA) and cluster analysis were performed, revealing excellent repeatability among the three replicates in the KBU2046-treated group (Fig. 3B–D). The top 20 proteins with the most significant changes are depicted in Fig. 3E.

Furthermore, we observed a down-regulation of genes such as LRRC8E, DNAL1, SLC39A1, MAFF, COL8A1, APEX1, NRIP1, GUCD1, UBE2E3, and LTBP3 in KBU2046-treated MDA-MB-231 cells, all of which are known to be associated with TGF-β1 activity [12], molecular motor function [17], the tumor microenvironment [18], proinflammatory cytokines and transcription factors [19], and cellular homeostasis [20] (Fig. 3E).

In the GO enrichment analysis, the differentially expressed proteins were implicated in various biological processes such as positive regulation of granulocyte-macrophage colony, positive regulation of inflammatory response, and positive regulation of membrane protein ectodomain (Fig. 3F). Subsequent KEGG enrichment analysis revealed the activation of the RNA polymerase pathway and motor proteins signaling pathway (Fig. 3G).

### KBU2046 decreases the expression of LRRC8E, LTBP3, MAFF, DNAL1 genes in vitro

**Figure 4:**
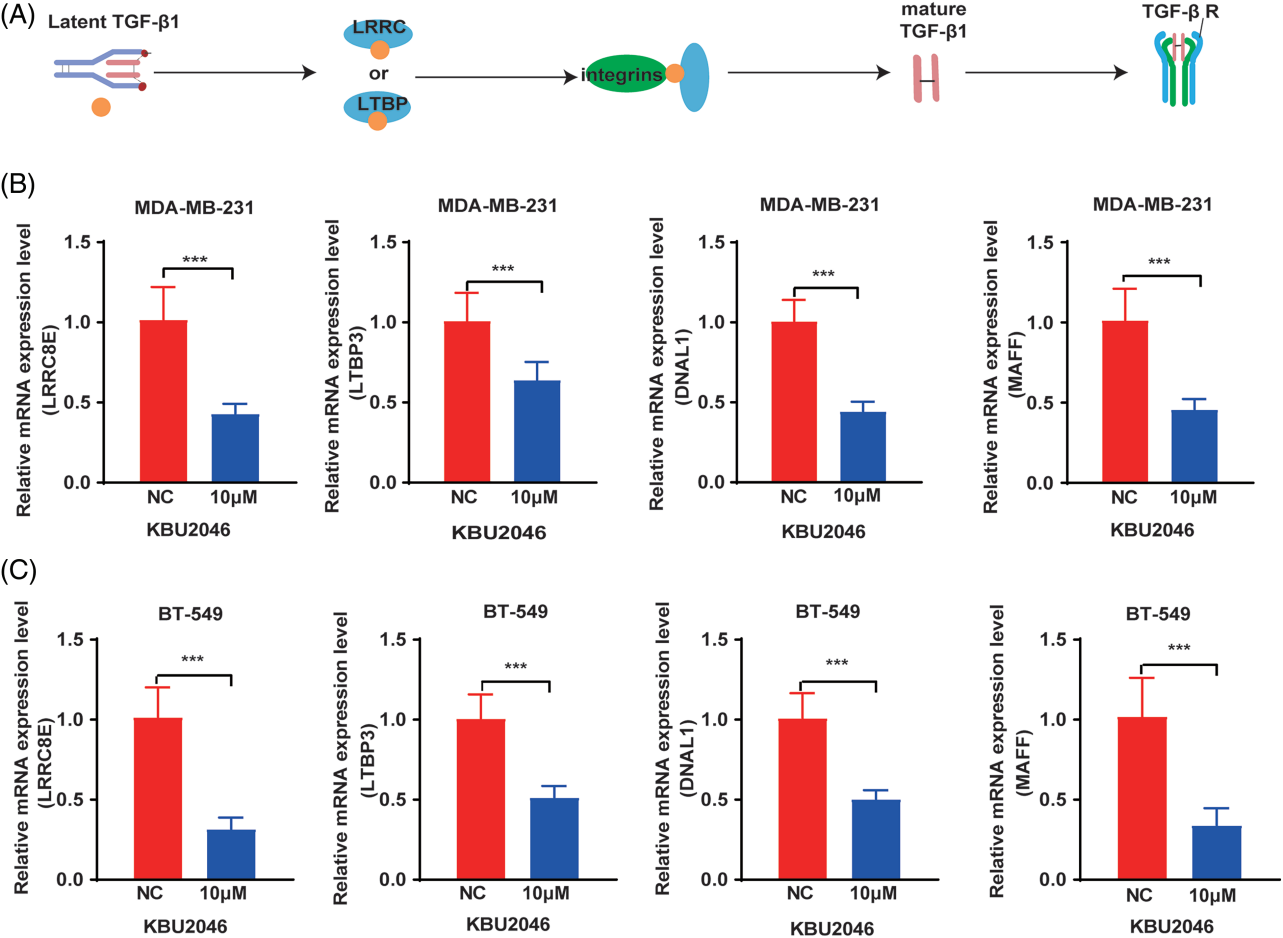
Differential gene expression in KBU2046-treated MDA-MB-231 and BT-549 cells. (A) Schematic diagram of key proteins activated by TGF-β1. (B) qRT-PCR was used to detect LRRC8E, LTBP3, DNAL1, MAFF gene expression in MDA-MB-231 cells. (C) qRT-PCR was used to detect LRRC8E, LTBP3, DNAL1, MAFF gene expression in BT-549 cells. ***p < 0.001.

To elucidate the molecular mechanisms by which KBU2046 attenuated TGF-β1 induced cellular motility, we investigated the differentially expressed genes identified through mRNA-seq analysis. Treatment with 10 μM KBU2046 in both MDA-MB-231 and BT-549 cells resulted in a significant reduction in the expression of LRRC8E, LTBP3, MAFF, and DNAL1 at the mRNA level, as revealed by qRT-PCR experiments (Fig. 4A–C, ***p < 0.001). LRRC8E and LTBP3 are crucial transmitters involved in the maturation of TGF-β1, serving as anchor proteins [12]. This finding implied that KBU2046 might play a pivotal role in modulating TGF-β1 activity.

### KBU2046 inhibits the expression of integrin αv and integrin α6 in TNBC and reverses the TGF-β1-mediated up-regulation of integrin αv and integrin α6

**Figure 5:**
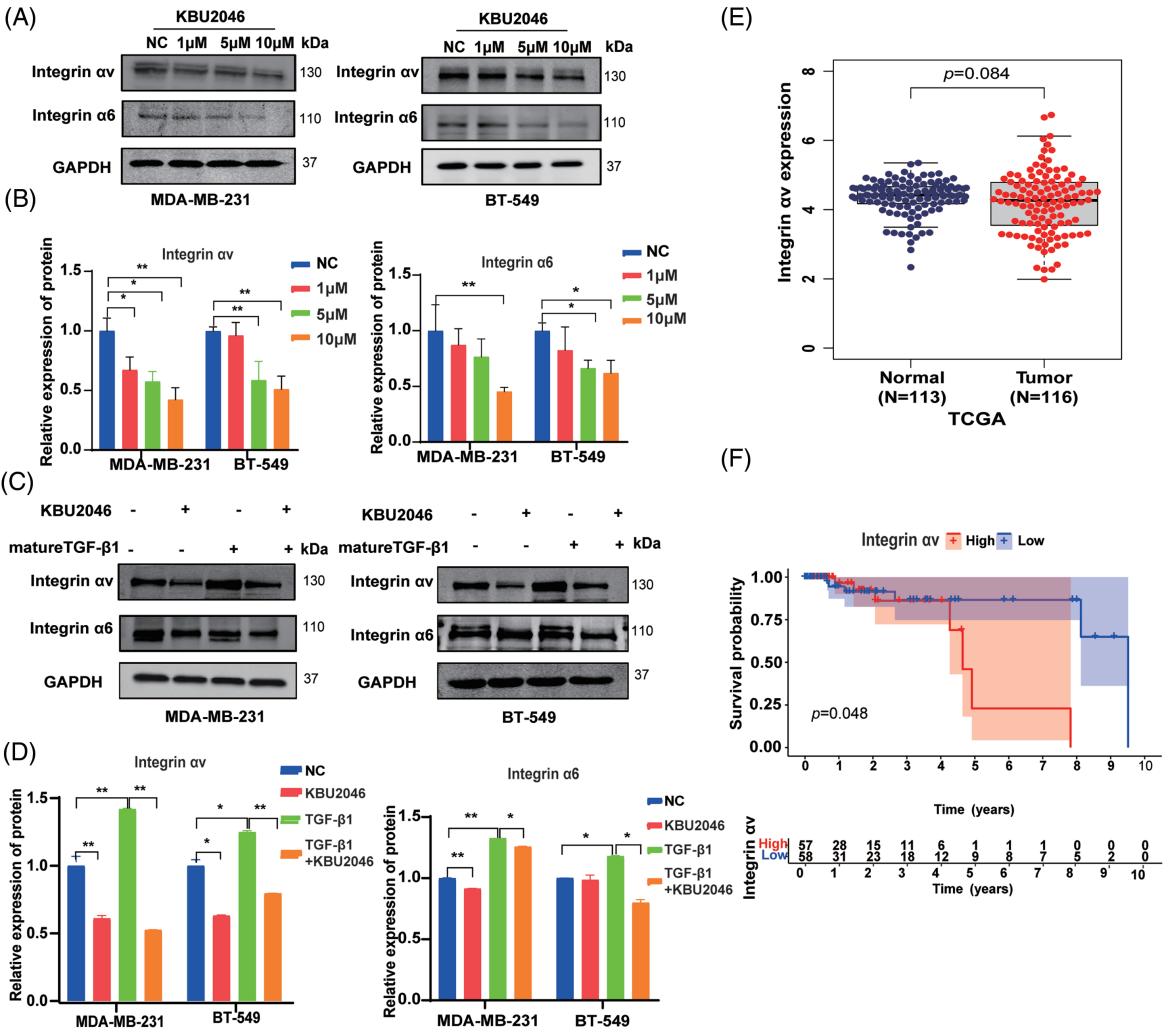
Effects of KBU2046 on integrin family in TNBC. (A and B) Effects of different concentrations of KBU2046 on the expression of integrin αv, integrin α6 in TNBC cells detected by Western blotting. (C and D) Effects of KBU2046 (10 µM) alone or in combination with TGF-β1 (10 ng/mL) on the expression of integrin αv and integrin α6 in TNBC cells detected by Western blotting. Data are presented as means ± SEM of at least three independent experiments. *p < 0.05; **p < 0.01; ns, no significance. (E and F) The expression and survival probability of integrin αv and in TNBC using the TCGA database.

To further validate the impact of KBU2046 on mature TGF-β1, we established *in vitro* TNBC cell models subjected to KBU2046 and mature TGF-β1 induction. Previous literature has indicated that integrins play a crucial role in the activation of latent TGF-β1 [21]. Consequently, we examined whether KBU2046 influenced members of the integrin family, specifically integrin αv, integrin α3, integrin α6, and integrin β8 proteins in TNBC cells (MDA-MB-231 and BT-549). The results demonstrated that KBU2046 could suppress the protein expression of integrin αv and integrin α6. In MDA-MB-231 cells treated with 1, 5, and 10 μM KBU2046, the corresponding protein levels of integrin αv were (67.28 ± 10.72)%, (57.61 ± 8.37)%, and (42.29 ± 9.97)%, and those values of integrin α6 were (87.15 ± 14.66)%, (76.54 ± 16.18)%, and (45.26 ± 3.93)%, respectively, compared to (100.00 ± 8.79)% and (100.00 ± 18.99)% in the negative control (Fig. 5A,B). Similarly, in BT-549 cells, protein expression levels of integrin αv were (96.13 ± 10.99)%, (58.68 ± 15.77)%, and (51.11 ± 10.92)%, and those values of integrin α6 were (82.47 ± 20.98)%, (66.29 ± 7.24)%, and (61.89 ± 11.58)%, respectively, compared to (100.00 ± 2.80)% and (100.00 ± 5.72)% in the negative control (Fig. 5A,B).

**Figure A1:**
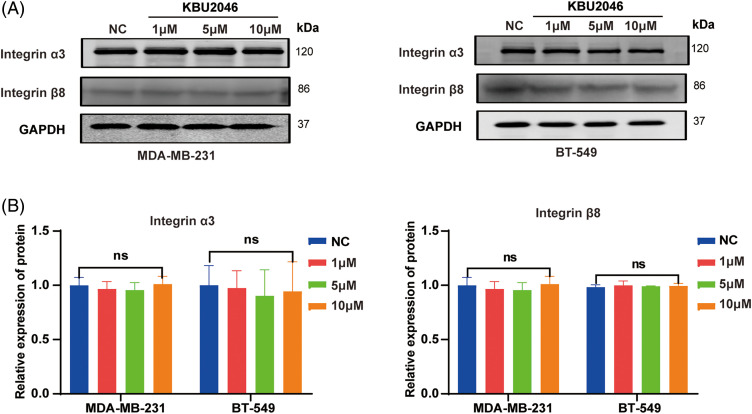
Effects of KBU2046 on integrin α3 and integrin β8 in TNBC. (A and B) Effects of different concentrations of KBU2046 on the expression of integrin α3, integrin β8 in TNBC cells detected by Western blotting. ns, no significance.

These results indicated that KBU2046 significantly suppressed the protein expressions of both integrin αv and integrin α6, with modest effects on the expressions of integrin α3 and integrin β8 (Fig. A1(A,B)). Furthermore, our findings revealed that mature TGF-β1 induced higher levels of integrin αv and integrin α6 proteins, while KBU2046 partially mitigated the TGF-β1-mediated enhancement of integrin αv and integrin α6 (*p* < 0.05) (Fig. 5C,D), suggesting that KBU2046 reduced TGF-β1 activation by targeting the integrin family.

### Integrin αv is up-regulated in TNBC and associated with poor prognosis

The aforementioned evidence suggested that KBU2046 suppressed the expression of integrin αv and integrin α6. Integrins αvβ6 and αvβ8 play specific roles in recognizing pro-TGF-β and activating its growth factor by releasing it from the latency imposed by its surrounding pro-domain [15]. We further assessed the expression of integrin αv in TNBC using the TCGA database. Our results indicated an up-regulation of integrin αv expression in the tumor group compared to the normal group from the TCGA database (Fig. 5E). Subsequently, we utilized normal and breast cancer data to generate a survival probability curve to analyze the prognostic value of integrin αv in TNBC. The results revealed a significant association between integrin αv expression level and survival probability in TNBC patients (*p* < 0.05, Fig. 5E,F). In summary, integrin αv was found to be up-regulated in breast cancer, and its expression was directly associated with poor survival rates in TNBC, suggesting that targeting integrin αv with KBU2046 might be a potential treatment strategy.

### KBU2046 inhibits Raf1 and ERK1/2 phosphorylation in TNBC

**Figure 6:**
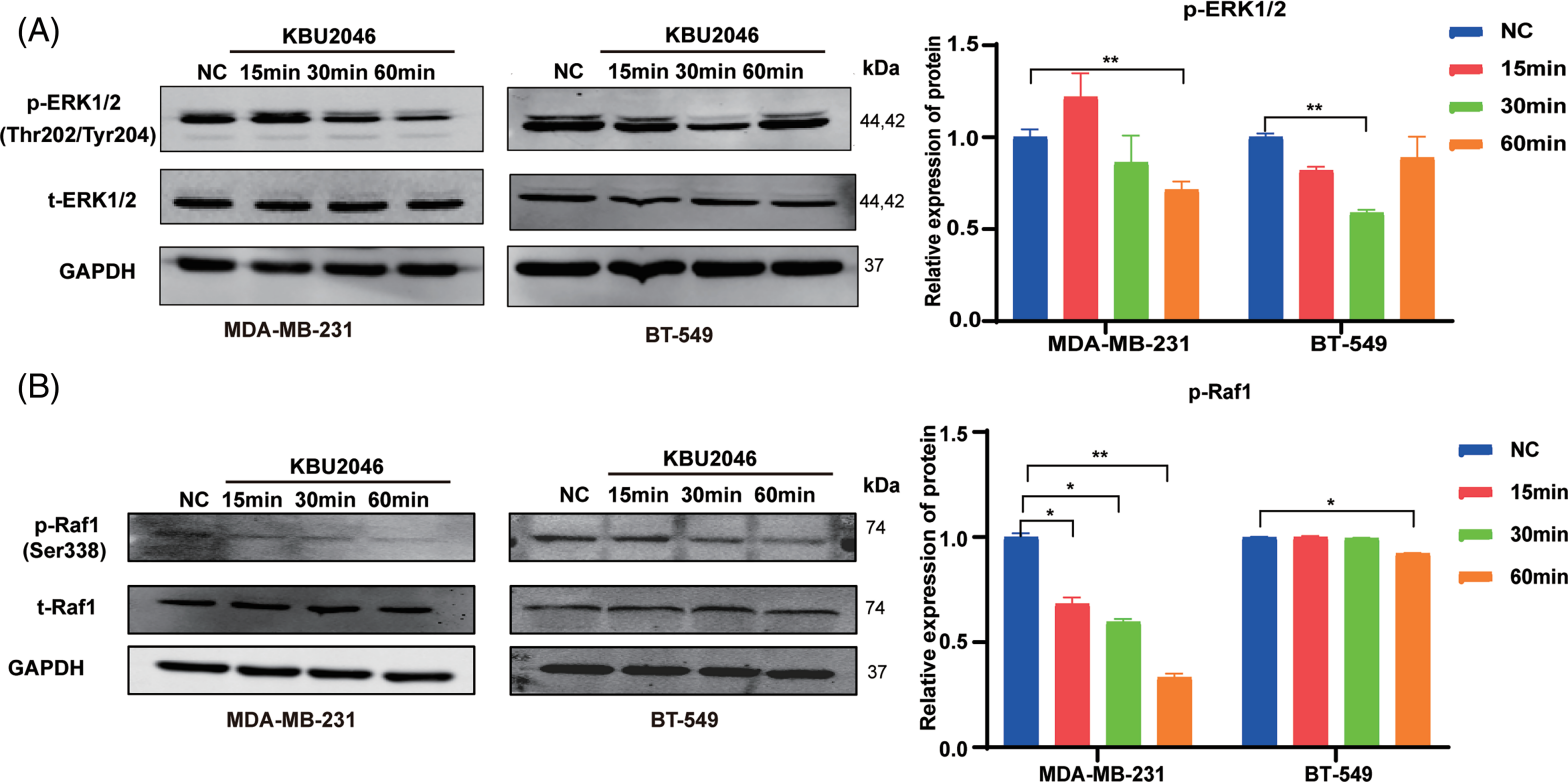
Effects of KBU2046 on MAPK signaling pathway in TNBC. (A) Expression levels of total ERK and p-ERK in TNBC cells treated with KBU2046 (10 µM) for different times detected by Western blotting analysis. (B) Expression levels of total Raf and p-Raf in TNBC cells treated with KBU2046 (10 µM) for different times were detected by Western blotting analysis. Data are presented as means ± SEM of at least three independent experiments. *p < 0.05; **p < 0.01.

Given KBU2046’s role in activating latent TGF-β1 complexes, we hypothesized that KBU2046 might affect TGF-β1 signaling. To further explore this mechanism, we examined the ability of KBU2046 to suppress Raf1 and ERK1/2 phosphorylation in breast cancer cells. Our results demonstrated that phosphorylation levels of Raf1 and ERK1/2 proteins treated with KBU2046 were significantly lower than those in the negative control (*p* < 0.05). KBU2046 robustly diminished Raf1 and ERK1/2 phosphorylation in MDA-MB-231 and BT-549 TNBC cells in a time-dependent manner (Fig. 6A,B). In conclusion, KBU2046 might inhibit Raf1 and ERK1/2 phosphorylation in TNBC, thereby partially regulating TNBC cell migration.

## Discussion

Breast cancer continues to be the most prevalent cancer among women globally [22]. TNBC is considered the most dangerous breast cancer subtype due to its accelerated progression, vast metastatic potential, and resistance to standard treatments [23,24]. While advanced multi-modal therapy has substantially improved the curability of early-stage breast cancer to 70%–80%, addressing advanced metastatic breast cancer remains a significant challenge [25,26]. TGF-β signaling is identified as a potential regulator of mammary epithelial cells associated with the risk of breast cancer [27].

In our present study, we observed that KBU2046 effectively inhibited TNBC cell motility without inducing cytotoxicity *in vitro*, specifically in MDA-MB-231 and BT-549 cell lines. These observations suggested that KBU2046 not only had the potential to mitigate tumor motility but also to counteract the effects induced by TGF-β1.

To elucidate the underlying mechanism of KBU2046 action in breast cancer cell lines, we conducted comprehensive mRNA-seq analysis and qRT-PCR. The results clearly demonstrated that KBU2046 down-regulated the expression of LRRC8E and LTBP3 at the mRNA level. In a context-specific manner, two major protein families, LTBPs and LRRC 32/33 proteins, play crucial roles in directly cross-linking with the latent form of TGF-β [28,29].

These findings collectively suggested that the anti-motility effect of KBU2046 was, at least in part, contingent upon the maturation of TGF-β1. This implied a potential therapeutic avenue where KBU2046 could effectively modulate breast cancer progression by targeting specific components of the TGF-β pathway. The nuanced insights provided by our study underscored the potential clinical significance of KBU2046 as a promising candidate for further investigation in the realm of breast cancer treatment.

To further elucidate the impact of KBU2046 on key downstream proteins during TGF-β1 activation, we investigated the expression of the integrin family in the KBU2046 treatment group. Our results unequivocally demonstrated that KBU2046 effectively inhibited the expressions of integrin αv and integrin α6 in TNBC cell lines. Notably, TNBC cells treated with KBU2046 exhibited a significant reduction in integrin αv and integrin α6 expressions compared to the negative control group.

Interestingly, TNBC cells treated with mature TGF-β1 demonstrated an increase in integrin αv and integrin α6 expressions, while the group treated with KBU2046 and TGF-β1 simultaneously exhibited a noteworthy reduction in integrin αv and integrin α6 expressions compared to the TGF-β1 treatment group. These observations suggested the presence of a positive feedback loop between integrin αv and mature TGF-β1.

In the context of cancer treatment, the inhibition of integrin αvβ8 has shown promise as a therapeutic approach [30]. Specifically, inhibiting the integrin αvβ8 protein in tumors has demonstrated effectiveness in targeting local immunosuppressive mechanisms that enable tumors to evade the immune system [31]. This is achieved by blocking the interaction between integrin αvβ8 on tumor cells and immune cells [31]. The potential regulatory role of KBU2046 in the tumor microenvironment unveils intriguing avenues for future studies, providing valuable insights into its broader therapeutic implications.

Moreover, reports suggest that normal oncogenic activation of the ERK pathway/MAPK/Ras/EGFR, in response to SMAD2/3 phosphorylation, also diminishes SMAD2/3 phosphorylation [32]. Robust activation of the Ras-ERK axis is pivotal for the transformation of TGF-β1 from a tumor suppressor gene to a tumor promoter gene [33]. In consideration of these insights, we tentatively posited that KBU2046 might exert anti-motility effects by regulating the Raf/ERK signaling pathways.

The limitations in our study included that further research is necessary to evaluate the function of KBU2046 *in vivo* and its potential effect on regulating the tumor microenvironment. Although immunotherapy highlighted cancer treatment in the past decade, relatively low efficacy rate and drug resistance need to be overcome. Our work may provide a potential strategy for future clinical regimens in TNBC because it inhibits the TGF-β1 activation of triple-negative breast cancer cells.

**Figure 7:**
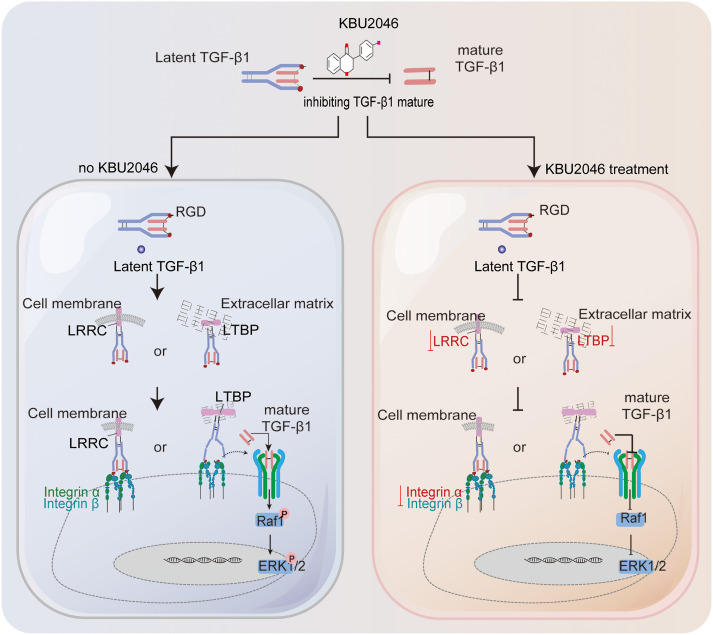
Schematic diagram illustrating the inhibition of KBU2046 on TNBC cell motility. The authors used Adobe Illustrator Artwork 27.0 to create the FIGURE 7 graph.

In summary, we proposed targeting TGF-β1 with KBU2046 as a potential therapeutic strategy to mitigate tumor motility and migration. This was accomplished by reducing the maturation of TGF-β1 and suppressing the TGF-β1/Ras/Raf1/ERK axis, as illustrated in Fig. 7. These findings offered valuable evidence for considering TGF-β1 as a biomarker and therapeutic target in breast cancer, highlighting the potential development of KBU2046 as a novel anticancer agent against breast cancer metastasis.

## Conclusion

Utilizing mRNA-seq and experimental results, we explored the key targets and signaling pathways responsible for KBU2046-mediated inhibition of TNBC metastasis. Our investigation revealed that KBU2046 effectively diminished TNBC motility by suppressing the activation of TGF-β1. These findings pinpointed TGF-β1 as a potential target for TNBC treatment using KBU2046 and provided theoretical support for its prospective preclinical application.

## Data Availability

The data that support the findings of this study are available on request from the corresponding author upon reasonable request.

## Appendix A

**Table A1:** Sequences of primer and probe

ID	Forward primers (5'-3')	Reverse primers (5'-3')
β-actin	CATGTACGTTGCTATCCAGGC	CTCCTTAATGTCACGCACGAT
LRRC8E	TACTTCCCTTACCTCGTGGTC	TGGAGAGTCGAAACACTTGCC
LTBP3	TCCCCAGGGCTACAAGAGG	AGACACAGCGATAGGAGCCA
DNAL1	GTCCATGCTTGCTAATTGCGA	AAGGGATTGCCTACAAACACC
MAFF	ATTCCGGGAAGCTCGCCTTA	CTGAGTTTTGTTGTGGGGCG
